# Mitochondrial bioenergetics dysfunction in T2DM: linking oxidative stress to insulin resistance

**DOI:** 10.3389/fendo.2025.1674477

**Published:** 2025-12-17

**Authors:** Zheng Feng, Zhenlin Tan, Donghui Lu

**Affiliations:** 1 Department of Endocrinology,Peking University ShenZhen Hospital, Shenzhen, China; 2 Shenzhen University Medicine School,, Shenzhen, Guangdong, China; 3 Faculty of Chinese Medicine, Macau University of Science and Technology, Taipa, Macao, China

**Keywords:** type 2 diabetes, insulin resistance, mitochondria, oxidative stress, electron transport chain, reverse electron transport, tricarboxylic acid cycle, delivery

## Abstract

Insulin resistance (IR) is a core pathological feature of type 2 diabetes mellitus (T2DM) and is closely associated with mitochondrial dysfunction in insulin-sensitive tissues, including skeletal muscle, liver, and adipose tissue. Mitochondrial abnormalities—such as impaired oxidative phosphorylation (OXPHOS), dysregulated tricarboxylic acid (TCA) cycle, excessive reactive oxygen species (ROS) production, and altered mitochondrial dynamics—can contribute to IR by oxidatively modifying insulin-signaling proteins and activating inflammatory pathways (JNK/NF-κB). Recent work also implicates microRNAs (miRNAs) as modulators that link mitochondrial function and redox balance to insulin action; however, their magnitude and tissue specificity in human T2DM remain to be defined. Therapeutic strategies that target mitochondrial bioenergetics and redox homeostasis show promise, while miRNA-directed approaches are emerging. This review provides an explanatory synthesis aimed at distinguishing associations within the mitochondria-ROS-insulin resistance axis supported by solid evidence from findings influenced by specific contexts, and outlines translational opportunities and their associated delivery bottlenecks.

## 1 Introduction

Mitochondrial energy metabolism dysfunction is a pivotal pathological feature in the pathophysiology of type 2 diabetes mellitus (T2DM), primarily characterized by decreased oxidative phosphorylation (OXPHOS) efficiency, disrupted tricarboxylic acid (TCA) cycle flux, elevated reactive oxygen species (ROS) production, and reduced mitochondrial quality (1). These bioenergetic abnormalities are widely observed in insulin-sensitive tissues, including skeletal muscle, liver, and adipose tissue, significantly impairing their responsiveness to insulin and contributing to systemic metabolic dysregulation (2–4). Extensive epidemiological and clinical evidence supports the critical role of mitochondrial dysfunction as both a cause and consequence of insulin resistance (IR) (3, 5, 6).

Excessive ROS accumulation not only damages mitochondrial structure but also disrupts the phosphorylation of key insulin signaling proteins and activates inflammatory pathways, including those modulated by adipokines such as resistin, thereby suppressing GLUT4 translocation and glycogen synthesis, and further exacerbating IR (7, 8). In this context, inflammation serves as a critical amplifying factor. Recent evidence suggests that diabetes induces “trained immunity” in neutrophils, priming them for increased neutrophil extracellular trap (NET) formation and systemic inflammatory reactivation, which may secondarily aggravate mitochondrial dysfunction and insulin resistance (9). This highlights a potential feedback loop wherein immune cell programming contributes to metabolic stress.

Not all studies agree on the extent, timing, or tissue distribution of mitochondrial deficits in insulin-resistant humans; rapid lipid-infusion models can induce IR without immediate mitochondrial changes, suggesting parallel or compensatory pathways. Accordingly, we distinguish established from emerging mechanisms and indicate where human evidence remains limited.

Despite substantial progress in understanding mitochondrial contributions to metabolic dysfunction, the precise causal relationships linking oxidative stress to mitochondrial impairments and insulin resistance remain incompletely elucidated. Notably, recent studies suggest that oxidative stress and mitochondrial dysfunction do not merely coexist but actively amplify each other, forming a self-perpetuating cycle that drives chronic metabolic inflammation and cellular dysfunction. For example, mitochondrial oxidative stress-induced senescence and inflammation have been increasingly recognized as interconnected mechanisms contributing to metabolic dysfunction. While these pathways have been extensively elucidated in cancer models, emerging evidence suggests that similar ROS-driven senescence–inflammation feedback loops may play a pathogenic role in chronic metabolic diseases such as T2DM, thereby amplifying tissue dysfunction and insulin resistance (10, 11). This feedback loop is particularly detrimental in tissues prone to microvascular damage and wound healing defects, where redox imbalance and mitochondrial distress impair regenerative responses and exacerbate diabetic complications (12–14).

In recent years, microRNAs (miRNAs) have garnered significant attention for their regulatory roles in mitochondrial homeostasis and ROS scavenging, emerging as critical molecular bridges between mitochondrial dysfunction and insulin resistance. MicroRNAs are short (~21–23 nt) non-coding RNAs that fine-tune gene expression post-transcriptionally, typically dampening rather than abolishing protein output across many targets per miRNA; they are conserved and context-dependent regulators.For instance, miR-29c has been reported to inhibit mitochondrial biogenesis through targeted downregulation of peroxisome proliferator-activated receptor gamma coactivator-1 alpha (PGC-1α) (15), while miR-126 modulates cellular antioxidant capacity by regulating the expression of antioxidant enzymes (16). Thus, systematically delineating the complex interaction mechanisms among mitochondrial bioenergetic dysfunction, oxidative stress, and insulin resistance—particularly through the lens of miRNA-mediated regulatory networks—is crucial for unveiling novel therapeutic targets. Such an integrative understanding may enable the development of innovative treatments aimed at restoring mitochondrial function, alleviating oxidative stress, and improving insulin sensitivity.

This review aims to elucidate how mitochondrial bioenergetics and oxidative stress collectively contribute to insulin resistance in insulin-sensitive tissues. We outline the limitations of evidence from human studies, highlight translational medicine levers such as treatment and delivery, and focus on the role of miRNAs as regulators. Recent research advances are summarized, therapeutic implications are discussed, unresolved issues are identified, and future research directions are proposed.

## 2 Search strategy and study selection

Records in PubMed, Web of Science, and Scopus (January 2010 to January 2025) were searched using the following combination of terms: type 2 diabetes, insulin resistance, mitochondria, oxidative stress/ROS, electron transport chain, reverse electron transport, tricarboxylic acid cycle, delivery. Studies included were those investigating mechanisms, translational and clinical research, as well as high-quality reviews; editorials and studies utilizing non-mammalian models with low relevance were excluded. Two authors independently screened titles/abstracts, followed by full-text review, prioritizing recent studies (published 2022–2024) that employed transparent methodologies and reported quantitative readouts.

## 3 Mitochondrial bioenergetics dysfunction in T2DM

### 3.1 Dysfunction of the mitochondrial electron transport chain

As the core of oxidative phosphorylation, ETC plays a pivotal role in energy metabolism. Its dysfunction is a primary contributor to metabolic disorders in insulin-sensitive tissues such as skeletal muscle and liver (17). In patients with T2DM, the activities of ETC complexes I and III are significantly reduced, leading to impaired electron transfer and abnormal reactions between electrons and oxygen, thereby catalyzing excessive generation of ROS such as superoxide anion (·O_2_^-^) (18–21). Specific mechanisms include reverse electron transfer at the complex I flavin mononucleotide (FMN) site, which is a cofactor for electron entry, and the accumulation of semiquinone radicals at the complex III quinone oxidation (Qo) site, both of which can directly produce reactive oxygen species. The Q_o_ site is an external quinol oxidation site on the intermembrane space side of the cytochrome bc_1_ complex (complex III), where ubiquinol is oxidized and can form a semiquinone intermediate, providing a recognized site for ROS generation (as opposed to the internal Qi site, where quinone is reduced).Clinical biopsies reveal that complex I activity in T2DM skeletal muscle is reduced by approximately 40%, showing a significant positive correlation with systemic insulin sensitivity (22). In the liver of obese T2DM patients, enhanced platelet-derived growth factor AA signaling contributes to insulin resistance (23). In the liver, PDGF-AA/PDGFRα signaling integrates matrix remodeling with hepatocyte insulin action; its upregulation in obesity is associated with inflammatory tension and fibrosis, providing a non-cell-autonomous cue that exacerbates hepatic insulin resistance. In adipose tissue, JNK activation links local inflammatory stress to proximal insulin signaling inhibition (multisite IRS-1 serine/threonine phosphorylation) and systemic impairment of glucose disposal (24).

Consequently, ETC damage breaks the normal cycle of energy metabolism through excessive ROS accumulation, thereby activating inflammatory kinases, blocking the binding of IRS-1 to insulin receptors, and even inducing mtDNA damage while inhibiting OXPHOS gene transcription (25–27). Furthermore, ROS oxidizes the AS160 protein, impairing GLUT4 translocation and glucose uptake (28). Therapeutic interventions targeting the ETC-ROS axis, such as metformin and resveratrol, have demonstrated efficacy in both clinical and experimental settings by reducing ROS levels and improving insulin sensitivity (5, 29, 30). Elevated mitochondrial ROS impairs insulin action through multiple nodes: activation of stress kinases (JNK/IKKβ), oxidation of lipid/kinase intermediates (ceramide/DAG-PKC), and mtDNA-triggered inflammatory pathways, which converge on IRS-1/Akt and GLUT4 trafficking. Nutrient excess and lipid overload enhance β-oxidation and excessively reduce the coenzyme Q pool, making complexes I/III prone to electron leakage. Pro-inflammatory signals and hypoxia elevate the mitochondrial membrane potentialand disrupt the supply–demand matching, further promoting ROS generation. Subsequently, oxidants damage mitochondrial DNA and supercomplex structures, shift mitochondrial dynamics toward fission, and inhibit the biosynthesis process mediated by PGC-1α/NRF1/TFAM—thereby forming a self-reinforcing cycle.Beyond mitochondrial ROS, ER stress/Unfolded Protein Response (UPR) (PERK/IRE1/XBP1) and lipid-induced DAG/PKC activation in muscle and liver constitute well-recognized routes to IR that can operate even when mitochondrial indices appear near-normal; these pathways likely interact with, rather than exclude, mitochondrial mechanisms.

The generation of ROS by Complexes I and III, with subsequent activation of the JNK/IKKβ signaling pathway, is a consistently observed phenomenon across different experimental models. In contrast, the relative importance of reverse electron transport under *in vivo* conditions is context-dependent, influenced by factors such as the reduction level of the ubiquinone pool and its re-oxidation rate. Heterogeneity in human data can be attributed to variations in tissue sampling, metabolic status, and analytical methodologies. From a clinical perspective, these redox-sensitive nodes are involved in the regulation of insulin-stimulated glucose uptake and hepatic glucose output. This provides a potential rationale for therapeutic interventions targeting redox signaling, while also underscoring the necessity for tissue-specific assessments.

### 3.2 TCA cycle dysregulation and accumulation of metabolic intermediates

Dysregulation of key enzymes in the TCA cycle leads to abnormal accumulation of metabolic intermediates, further disrupting insulin signaling (31). Citrate synthase (CS) activity is reduced by about 10–30% in the skeletal muscle of T2DM patients (32, 33), hindering acetyl-CoA entry into the TCA cycle and diminishing NADH production, TCA-driven NADH decreases constrain electron supply to Complex I (substrate limitation) and lower flux. Moreover, insufficient citrate synthesis diverts substrate flux toward the succinate pathway, while succinate dehydrogenase (SDH) is oxidized by ROS, resulting in intracellular succinate accumulation (34). Elevated succinate levels have been detected in the urine of diabetic patients, and succinate is now considered a potential biomarker for metabolic disorders. However, the precise mechanism by which it affects insulin signaling remains to be elucidated (35). Under conditions of succinate accumulation, particularly during re-oxidation, reverse electron transfer (RET) occurs at Complex I (36). RET, the backward flow of electrons from the highly reduced ubiquinol (QH2) pool through complex I to NAD^+^, is facilitated by the high membrane potential (Δψm) and succinate-driven reduction through complex II; RET is a potent context-dependent source of ROS in complex I. RET-driven ROS at Complex I is a context-dependent amplifier (e.g., rapid re-oxidation after succinate accumulation) but not universally dominant; forward leak at Complex I and semiquinone at Complex III also contribute. RET occurs primarily under conditions of mitochondrial dysfunction when the Q-pool becomes over-reduced. In insulin-resistant states, RET is facilitated by impaired OXPHOS, and is particularly prominent during conditions of re-oxygenation or metabolic stress. Under these conditions, the accumulation of succinate and the inability to oxidize it properly lead to an over-reduction of the Q-pool, which drives RET at Complex I. The resulting ROS generation further disrupts mitochondrial function and contributes to insulin resistance. This reinforces the vicious cycle of mitochondrial stress and metabolic dysfunction commonly observed in T2DM. Succinate is an electron donor to Complex II; elevated succinate typically reflects altered II→III flux/Q-pool over-reduction rather than direct Complex III inhibition. This ROS burst can lead to the inhibition of IRS/Akt phosphorylation. In summary, SDH is a key enzyme in the mitochondrial electron transport chain, responsible for catalyzing the conversion of succinate to fumarate. However, ROS-induced SDH impairment compromises its ability to oxidize succinate, leading to succinate accumulation. The buildup of succinate maintains the Q pool in an overly reduced state, which in turn triggers RET at complex I. This process results in increased ROS production, thereby exacerbating mitochondrial dysfunction and worsening insulin resistance. Succinate oxidation via SDH is essential for maintaining the redox balance of the Q pool. When this pathway is inhibited, succinate accumulation further aggravates oxidative stress.

**Figure 1:**
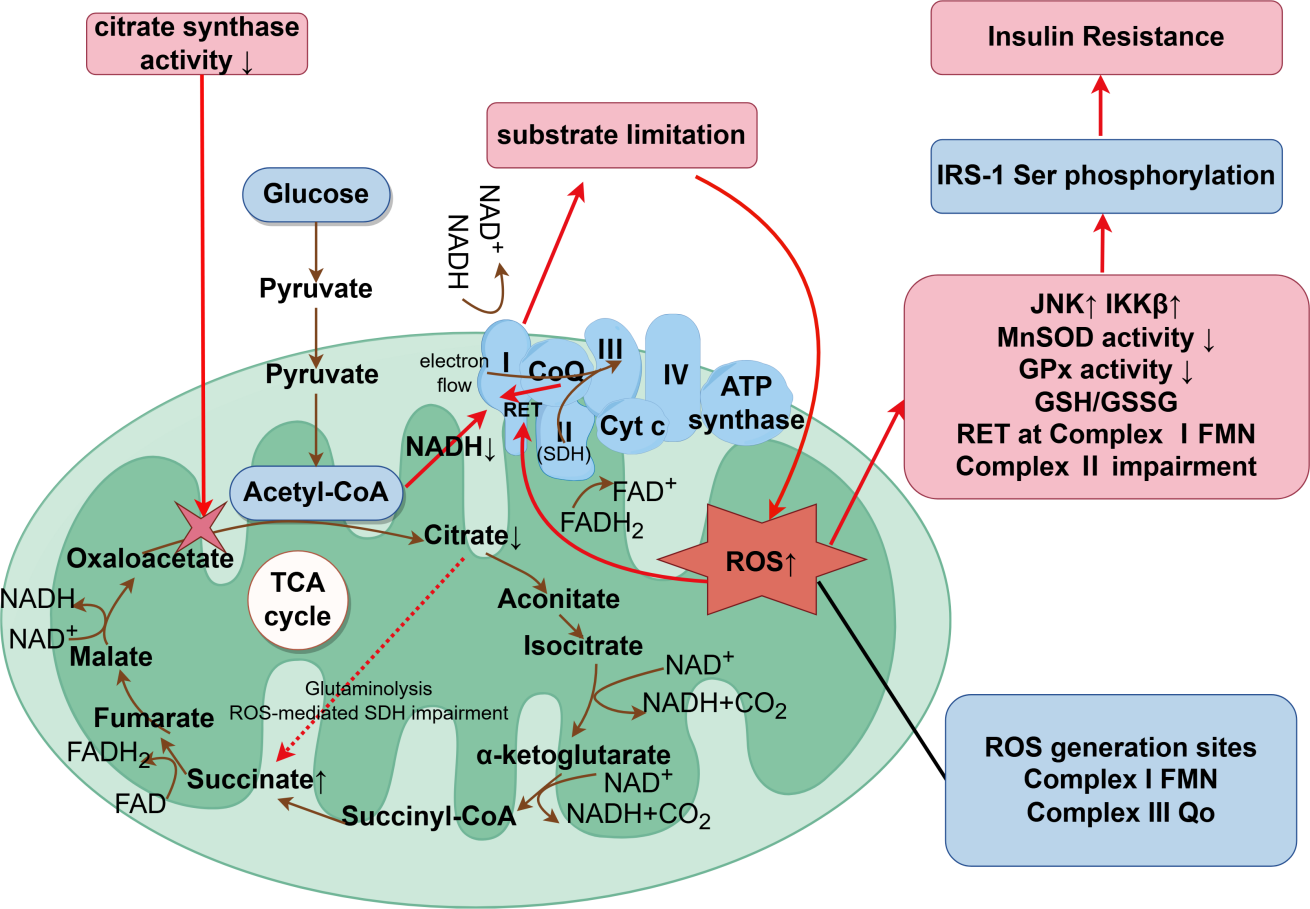
Dysregulated tricarboxylic acid cycle flux and succinate-driven redox imbalance in T2DM. CS activity is reduced, limiting the entry of acetyl-CoA and the generation of reduced NADH, thereby slowing TCA cycle flux. Succinate accumulates due to decreased succinate dehydrogenase (SDH, Complex II) activity and ROS-mediated inhibition of SDH. Under normal conditions, succinate oxidation reduces the ubiquinone pool (CoQ/CoQH₂); when SDH function is impaired, the ubiquinone pool becomes over-reduced due to continued electron input from residual β-oxidation. The over-reduced ubiquinone pool promotes RET at the FMN site of Complex I, resulting in substantial ROS production. Complex III (Qo site) also contributes to this process via semiquinone-derived ROS. Excess ROS triggers JNK/IKKβ activation, leading to IRS-1 serine phosphorylation, which subsequently impairs the IRS-1/Akt/GLUT4 signaling pathway, ultimately establishing a self-reinforcing oxidative-metabolic cycle in insulin-sensitive tissues.

In insulin-sensitive tissues such as skeletal muscle, elevated mitochondrial ceramide is a necessary and often sufficient upstream event that induces IR. This occurs through disruption of respirasome stability and depletion of coenzyme Q (CoQ), leading to OXPHOS-ROS imbalance and downstream suppression of IRS-1/Akt signaling. CoQ depletion is independently associated with IR and serves as a key mediator in the aforementioned pathway. Supplementation with CoQ can counteract ceramide-induced IR, whereas reducing ceramide alone is insufficient to fully reverse established CoQ deficiency (37, 38). Overall, the ceramide-CoQ-ROS axis forms a self-reinforcing pathological loop, representing a potential target for combined therapeutic intervention. The IRS/Akt pathway is a critical insulin signaling cascade, and suppression of its phosphorylation typically indicates aggravated insulin resistance (39, 40). Although downregulating CS reduces TCA cycle flux, when β-oxidation remains elevated, it exacerbates redox imbalance, leading to excessive reduction of CoQ and increased electron leakage at Complexes I/III; the reduction in biogenesis/supercomplex stability further elevates the probability of ROS generation.Animal studies in mice models have demonstrated that elevated succinate levels may impair mitochondrial complex I-III activity, thereby reducing ATP production (41). Furthermore, an abnormal citrate/isocitrate ratio can activate cytosolic ATP-citrate lyase (ACLY), whose expression is inversely correlated with the degree of insulin resistance (42). In summary, dysregulation of the TCA cycle establishes a vicious cycle of “TCA metabolites-insulin signaling suppression,” further exacerbating insulin resistance. This mechanism is illustrated in **Figure 1**, highlighting how TCA cycle disruption and succinate accumulation contribute to ROS-driven insulin resistance via RET.

CS activity is reduced, limiting the entry of acetyl-CoA and the generation of reduced NADH, thereby slowing TCA cycle flux. Succinate accumulates due to decreased succinate dehydrogenase (SDH, Complex II) activity and ROS-mediated inhibition of SDH. Under normal conditions, succinate oxidation reduces the ubiquinone pool (CoQ/CoQH_2_); when SDH function is impaired, the ubiquinone pool becomes over-reduced due to continued electron input from residual β-oxidation. The over-reduced ubiquinone pool promotes RET at the FMN site of Complex I, resulting in substantial ROS production. Complex III (Qo site) also contributes to this process via semiquinone-derived ROS. Excess ROS triggers JNK/IKKβ activation, leading to IRS-1 serine phosphorylation, which subsequently impairs the IRS-1/Akt/GLUT4 signaling pathway, ultimately establishing a self-reinforcing oxidative-metabolic cycle in insulin-sensitive tissues.

### 3.3 Imbalance in mitochondrial fission/fusion dynamics

Mitochondria maintain structural and functional homeostasis through dynamic fission and fusion, a process tightly regulated by GTPases (43). Dynamin-related protein 1 (Drp1) is a key mediator of mitochondrial fission, whose activity is modulated by phosphorylation: phosphorylation at Ser616 promotes its recruitment to the outer mitochondrial membrane, inducing fission, whereas phosphorylation at Ser637 inhibits its activity (44–46). In T2DM or insulin-resistant states, Drp1 is aberrantly activated, leading to excessive mitochondrial fragmentation (47–50). Studies in animal models demonstrate that high-fat diet-induced T2DM mice exhibit increasion in Drp1 Ser616 phosphorylation in skeletal muscle, accompanied by a significant rise in mitochondrial fragmentation (51). Structural disruption directly impairs mitochondrial function: cristae disassembly destabilizes ETC supercomplexes, promoting electron leakage and O_2_^-^ generation (51). Mitochondrial fission facilitates PINK1–Parkin-dependent mitophagy by segregating damaged segments; excess fission without efficient mitophagy accumulates dysfunctional mitochondria and heightens ROS (52). Activation of the miR-30–p53–DRP1 axis by the miR-30 family can induce severe mitochondrial structural damage (53). A closed loop of mitochondrial fragmentation–ROS accumulation–insulin signaling blockade is established: ROS activates the JNK/p38 MAPK pathway, inducing IRS-1 Ser307 phosphorylation and inhibiting its interaction with the insulin receptor (54). Additionally, ROS suppresses pyruvate dehydrogenase (PDH) activity, blocking acetyl-CoA entry into the TCA cycle and further exacerbating energy metabolism dysfunction (55). Clinical studies indicate that miR-30a-5p, miR-30d-5p, and miR-30c-5p are involved in glucose metabolism and insulin signaling, making them promising biomarkers for future T2DM research (56). The Drp1 inhibitor Mdivi-1 has demonstrated potential in improving glucose metabolism abnormalities in experimental models (57, 58).

## 4 Oxidative stress: a bridge between mitochondria and IR

### 4.1 Sources and molecular targets of ROS

This section integrates how mitochondrial ROS participate in stress kinase signaling, mitochondrial DNA signaling, and the antioxidant network, based on the mechanics of the ETC and the TCA cycle, and links these molecular events to tissue-level insulin action.

Low-level mitochondrial ROS act as signaling (oxidative eustress) and can facilitate insulin action, whereas chronic, dysregulated ROS cause damage (oxidative distress). Our focus is on chronic distress and restoring redox balance—not abolishing ROS—which explains why therapies aim to modulate ROS or boost endogenous defenses. Oxidative stress plays a pivotal role in the development of IR, primarily driven by electron leakage from the mitochondrial ETC and activation of NADPH oxidase (NOX) (59). ·O_2_^-^, as the primary ROS, is overproduced at mitochondrial complexes I and III under IR conditions (60), impairing insulin signaling through the following mechanisms: (1) Stress Kinase-Mediated Serine Phosphorylation of IRS-1. ROS activates multiple stress-responsive kinase cascades, thereby altering the phosphorylation status of insulin signaling intermediates. O_2_^-^ activates the ASK1–MKK7–JNK axis, leading to IRS-1 Ser307 phosphorylation, which blocks its binding to the insulin receptor (61). O_2_^-^ promotes IKKβ activation, enhancing IRS-1 Ser312 phosphorylation while inducing the release of pro-inflammatory cytokines, thereby establishing a positive feedback loop between inflammation and oxidative stress (62). (2) Signal Amplification via Oxidative Damage to Mitochondrial DNA. Due to their close proximity to ROS generation sites and lack of histone protection, mitochondrial DNA (mtDNA) is highly vulnerable to oxidative damage (63). Superoxide anions induce the formation of 8-hydroxy-2’-deoxyguanosine (8-OHdG), increasing mtDNA mutation rates and impairing the transcription of respiratory chain components such as ND1 and ND6 (64). Damaged mtDNA released into the cytoplasm activates the cGAS–STING innate immune signaling pathway, further amplifying IKKβ and JNK activation and perpetuating a chronic inflammatory state through the “ROS–mtDNA–inflammation” axis (65). (3) ROS-Induced Immune-Metabolic Crosstalk and PANoptosis. In addition to direct disruption of metabolic signaling, emerging evidence reveals that oxidative stress can trigger programmed inflammatory cell death (PANoptosis)—a convergence of apoptosis, necroptosis, and pyroptosis—via activation of the tumor suppressor p53. In diabetic tissues, persistent ROS elevation upregulates p53 signaling, promoting mitochondrial damage, cytokine release, and immune cell infiltration, which collectively exacerbate metabolic inflammation and insulin resistance (66). This p53–PANoptosis axis provides a mechanistic bridge linking oxidative stress to immune-metabolic dysfunction in diabetes. Furthermore, chronic hyperglycemia and ROS imbalance are increasingly recognized to impair innate immunity and increase susceptibility to infections such as tuberculosis, indicating a broader systemic impact of metabolic oxidative stress on immunological competence (67).We refer to murine IRS1 Ser307 (human Ser312) and murine Ser612 (human Ser616) where specified; some knock-in studies show that mutation of Ser307 does not protect from IR and that multiple serine sites are insulin-responsive under physiological conditions, underscoring the complexity of “proximal signaling defect” models. Not all groups find a primary proximal defect in human obesity/T2DM, indicating heterogeneity across tissues and contexts (68, 69).The traditional focus on proximal IRS1–PI3K–Akt defects is increasingly balanced by evidence that insulin resistance often reflects downstream disturbances in GLUT4 trafficking and lipid handling, with no consistent defect at the proximal nodes (70). Thus, mitochondrial ROS may impair insulin action through multiple nodes, not exclusively via IRS1 serine phosphorylation.Lin et al. demonstrated in mice that lotus seedpod extract reduces ROS production, and this effect was mediated by increased phosphorylation of IRS-1 (71); Liu et al. showed in cell lines that the ROS-lowering effect of Salvia miltiorrhiza Bge was achieved through reduced phosphorylation of IRS-1/Akt (72). Further validation of this mechanism in humans is still required.

### 4.2 Dysfunction of the antioxidant defense system

Impaired antioxidant systems constitute a critical mechanism underlying the progressive exacerbation of oxidative stress in IR, primarily involving the following aspects. Manganese superoxide dismutase (MnSOD): Elevated superoxide anion concentrations significantly reduce MnSOD activity and markedly inhibit its catalytic efficiency (73). Glutathione Peroxidase (GPx): The activity of selenium-dependent GPx is compromised by hydrogen peroxide (H2O2) accumulation and glutathione (GSH) depletion, with a decreased GSH/GSSG ratio observed under insulin resistance (IR) conditions (74, 75). Nuclear factor erythroid 2-related factor 2 (NRF2), a key transcription factor regulating antioxidant response element (ARE) activity, is inhibited through multiple mechanisms. KEAP1-dependent degradation: ROS enhance the activity of the KEAP1–Cullin3 E3 ubiquitin ligase complex, promoting NRF2 ubiquitination and subsequent proteasomal degradation, thereby limiting the transcriptional activation of antioxidant genes (76). miR-500a-5p-mediated post-transcriptional regulation: In diabetic patients, upregulated miR-500a-5p suppresses NRF2 translation by directly binding to the 3’ untranslated region (3’ UTR) of Nrf2 mRNA, leading to diminished antioxidant enzyme expression (77). Epigenetic repression of NRF2 signaling: In addition to post-translational and post-transcriptional mechanisms, recent findings suggest that NRF2 can also be modulated at the epigenetic level. For example, studies have found that scutellarin can alleviate oxidative stress and cellular senescence in bone marrow mesenchymal stromal cells under diabetic conditions by activating the Ezh2–NRF2 axis. Cellular senescence denotes irreversible growth arrest accompanied by upregulation of p16 and p21, persistent DNA-damage responses, remodeling of cellular architecture and metabolism, and emergence of the senescence-associated secretory phenotype. This mechanism supports the therapeutic potential of targeting the NRF2 signaling pathway to restore redox homeostasis in tissues affected by type 2 diabetes (78). When NRF2 is inactivated, expression of key antioxidant enzymes including HO-1 and NQO1 declines, leading to impaired removal of reactive oxygen species (79). Concurrently, diminished NRF2 activity attenuates its inhibitory effect on NF-κB (80), exacerbating pro-inflammatory cytokine-induced insulin signaling blockade and forming a “oxidative stress–inflammation–insulin resistance” positive feedback loop. It is worth mentioning that KEAP1-Cullin3 can regulate other substrates other than NRF2, so the NRF2-centric explanation should take into account the potential network-level impact.

## 5 MicroRNAs in the mitochondria–ROS–insulin resistance axis

Dysregulated microRNAs (miRNAs) modulate mitochondrial biogenesis, redox homeostasis, and insulin signaling in T2DM. miR-141/200c repress SIRT1 and thereby blunt PGC-1α deacetylation/activation, attenuating mitochondrial biogenesis and antioxidant support programs (81–84). miR-34a is elevated in pancreatic islets and promotes β-cell vulnerability by targeting BCL-2 and reducing SOD2, which increases H_2_O_2_ and drives apoptosis; together, these effects aggravate tissue oxidative stress and insulin resistance (85–88).

**Figure 2:**
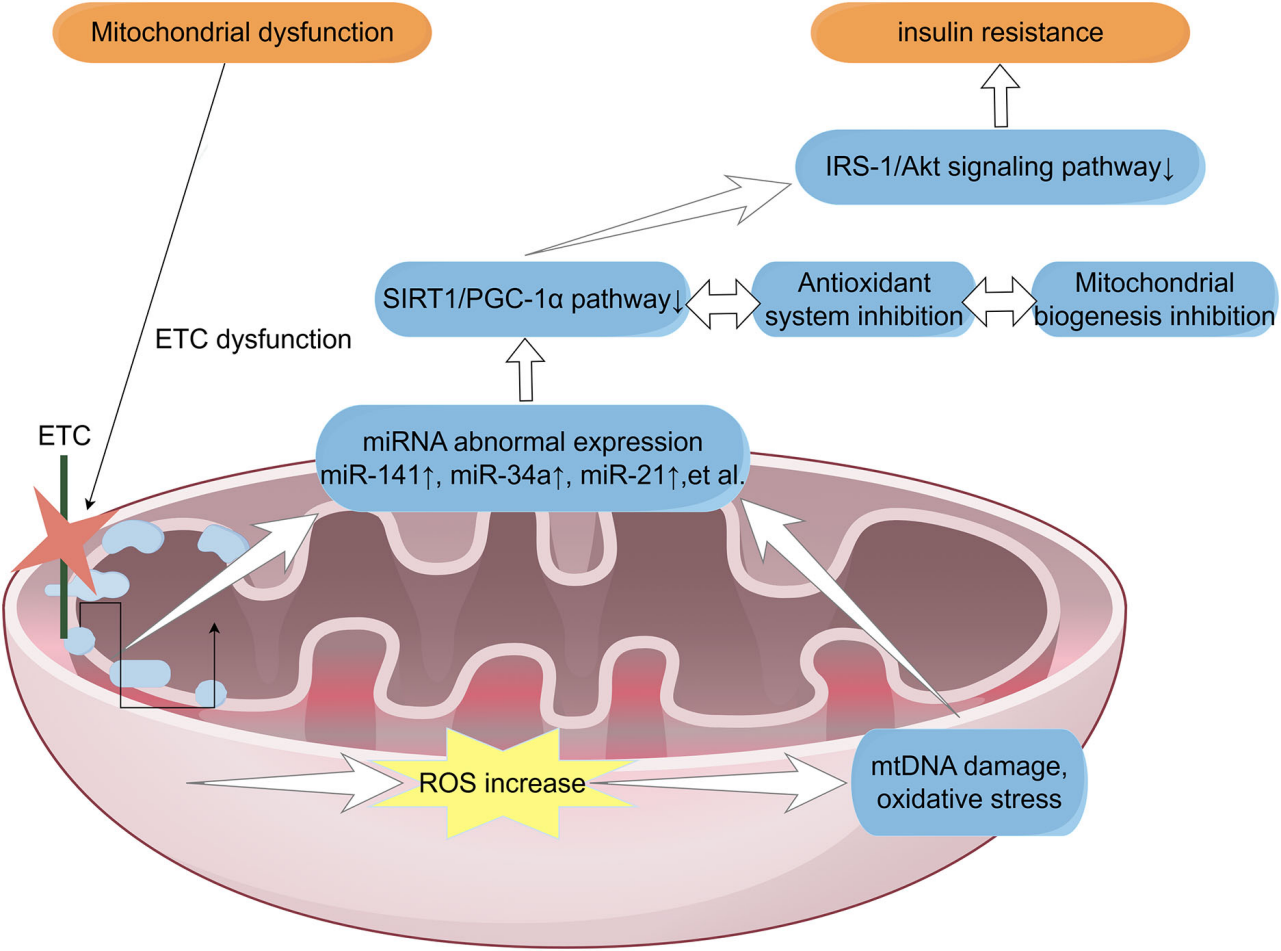
MiRNAs as regulators connect modules of mitochondrial biogenesis, redox control, and insulin signaling in T2DM. ETC dysfunction can lead to elevated ROS and ultimately insulin resistance, in which miRNAs play a role as regulators; major ROS sites include complex I (FMN/entry zone) and complex III (Qo/ubiquitinone). miR-141/200c → SIRT1↓ → PGC-1α activity↓ attenuates mitochondrial biogenesis; PGC-1α indirectly supports antioxidant capacity through NRF1/TFAM and FOXO3a coactivation [82–85]. miR-34a targets BCL-2 and SOD2, increasing β-cell apoptosis and oxidative stress [86–89]. miR-21 (PTEN→AKT) acutely enhances NRF2/ARE, but chronic AKT→mTOR/S6K may inhibit proximal insulin signaling [90–94]. miR-126 limits NOX4 and supports endothelial/oxidative homeostasis [95–97]; miR-375 regulates PDK1→PDH flux [98]. let-7 (LIN28/let-7 axis) dampens INSR/IRS2/IGF1R/AKT2, reducing PI3K–Akt signaling and glucose uptake; miR-33a/b (SREBF-embedded) repress CPT1A/PGC-1α/AMPKα/SIRT6 and ABCA1/ABCG1, limiting fatty-acid β-oxidation/mitochondrial biogenesis and lipid effluxThese miRNA nodes converge on IRS-1/Akt and GLUT4 trafficking. See Table 1 for organizational background, primary objectives, and functional readouts.

miR-21 shows context-dependent effects: by suppressing PTEN it elevates AKT and can acutely enhance NRF2/ARE responses, whereas chronic AKT→mTOR/S6K signaling may impair proximal insulin signaling and favor insulin resistance (40, 89–92). In the vasculature, miR-126 targets NOX4, aligns with lower NADPH-oxidase activity, and correlates with systemic antioxidant status (SOD increasing, MDA decreasing) (93–95). miR-375 suppresses PDK1 to enhance PDH flux and is variably detected in circulation, with tissue-specific implications for β-cell function (96). From a therapeutic perspective, antagonizing miR-34a improves β-cell survival in preclinical models (97).Clinical studies have demonstrated that the upregulation of let-7 is associated with an increased risk of type 2 diabetes mellitus (T2DM). However, the specific mechanisms of let-7 in T2DM remain unclear. Ex vivo studies have shown that let-7 can influence glucose metabolism via the PI3K–Akt pathway (98, 99). In preclinical models, aerobic exercise and vitamin D supplementation have been shown to improve lipid metabolism and insulin resistance in T2DM models by modulating hepatic miR-33 expression (100, 101).We separate well-supported processes, namely ROS production at Complex I and III and stress-kinase signaling, from provisional mechanisms such as *in-vivo* RET amplification and miR-29 or miR-141 regulation of PGC-1α. The majority of miRNA data, including studies on miR-34a and miR-21, derive from animal or *in-vitro* systems and require validation in humans. **Figure 2** summarizes the pathological cascade involving mitochondrial dysfunction, ROS accumulation, and dysregulated miRNA modulation converging on the IRS-1/Akt signaling pathway.Key miRNAs implicated in the mitochondria–ROS–insulin resistance.Key miRNAs associated with mitochondria-ROS-insulin resistance were described in **Table 1**.

**Table 1:** Key miRNAs implicated in the mitochondria–ROS–insulin resistance axis.

miRNA	Tissue/context	Primarytargets/pathways	DirectioninT2DM (ormetabolicdisease)	Functionalreadouts
miR-141	Cardiomyocytes (H/R), obesity; (evidence in insulin-sensitive tissues of T2DM is limited)	SIRT1 ↓ → impaired mitochondrial dynamics (MFN2)	↑ in obesity; ↑ under H/R stress	↓SIRT1/MFN2;Obesity association studies suggest that SIRT1 (T2DM can be inhibited in human tissues with limited direct evidence (80–83)
miR-34a	Pancreatic islets, liver/adipose	Bcl-2 ↓ (β-cell apoptosis); NAMPT/NAD^+^/SIRT1 axis ↓	↑ in obesity/diabetes models	↑ β-cell apoptosis; ↓ NAD^+^ and SIRT1 activity; correlated with IR/fatty liver (84–87)
miR-21	Endothelium, kidney/liver (diabetic complications)	PTEN→PI3K/AKT activation; context-dependent crosstalk with NRF2/ARE	↑ in hyperglycemia/DR and other models	↑AKT (and associated with NRF2/ARE antioxidant transcription in multiple models) (89–92)
miR-126	Endothelium; circulating (plasma/serum)	Vascular homeostasis; links to NOX/ROS signaling and endothelial integrity	↓ in diabetic vasculature/plasma	Downregulation is associated with increased endothelial ROS, inflammation, and impaired vascular function; It is one of the commonly used cyclic markers (40,93, 94)
miR-375	Pancreatic β-cells; circulation (variable)	PDK1 → PDH	↑ (Pancreatic islets), variable in plasma	Direct targeting PDK1
let-7 family	Liver, skeletal muscle, adipose (LIN28/let-7 axis)	INSR, IRS2, IGF1R, AKT2 → dampened PI3K–Akt signaling; suppression of glucose uptake and insulin responsiveness; adipose browning reduced	↑	↓ insulin signaling/GLUT4 translocation, ↑ hepatic gluconeogenesis, IR aggravated; anti-let-7 or LIN28 activation improves glucose tolerance/insulin sensitivity (context-dependent; strongest in preclinical models)
miR-33 (a/b)	Liver, adipose, macrophage (encoded in SREBF2/SREBF1)	CPT1A, PPARGC1A/PGC-1α, PRKAA1/AMPKα, SIRT6 (fatty-acid β-oxidation/mitochondrial biogenesis); ABCA1/ABCG1 (lipid efflux)	↑ (metabolic syndrome/NAFLD contexts)	↑ lipid accumulation/steatosis, IR worsened; anti-miR-33 improves insulin sensitivity and HDL short-term

Collectively, miRNAs regulate mitochondrial biogenesis, antioxidant defense, and insulin signaling, positioning them as regulatory nodes within the mitochondrial–ROS–IR axis; their tissue-specific roles and effect sizes in humans require further validation, which we prioritize for future investigation.

ETC dysfunction can lead to elevated ROS and ultimately insulin resistance, in which miRNAs play a role as regulators; major ROS sites include complex I (FMN/entry zone) and complex III (Qo/ubiquitinone). miR-141/200c → SIRT1↓ → PGC-1α activity↓ attenuates mitochondrial biogenesis; PGC-1α indirectly supports antioxidant capacity through NRF1/TFAM and FOXO3a coactivation (81–84). miR-34a targets BCL-2 and SOD2, increasing β-cell apoptosis and oxidative stress (85–88). miR-21 (PTEN→AKT) acutely enhances NRF2/ARE, but chronic AKT→mTOR/S6K may inhibit proximal insulin signaling (40, 89–92). miR-126 limits NOX4 and supports endothelial/oxidative homeostasis (93–95); miR-375 regulates PDK1→PDH flux (96). let-7 (LIN28/let-7 axis) dampens INSR/IRS2/IGF1R/AKT2, reducing PI3K–Akt signaling and glucose uptake; miR-33a/b (SREBF-embedded) repress CPT1A/PGC-1α/AMPKα/SIRT6 and ABCA1/ABCG1, limiting fatty-acid β-oxidation/mitochondrial biogenesis and lipid effluxThese miRNA nodes converge on IRS-1/Akt and GLUT4 trafficking. See **Table 1** for organizational background, primary objectives, and functional readouts.

## 6 Therapeutic implications

**Figure 3:**
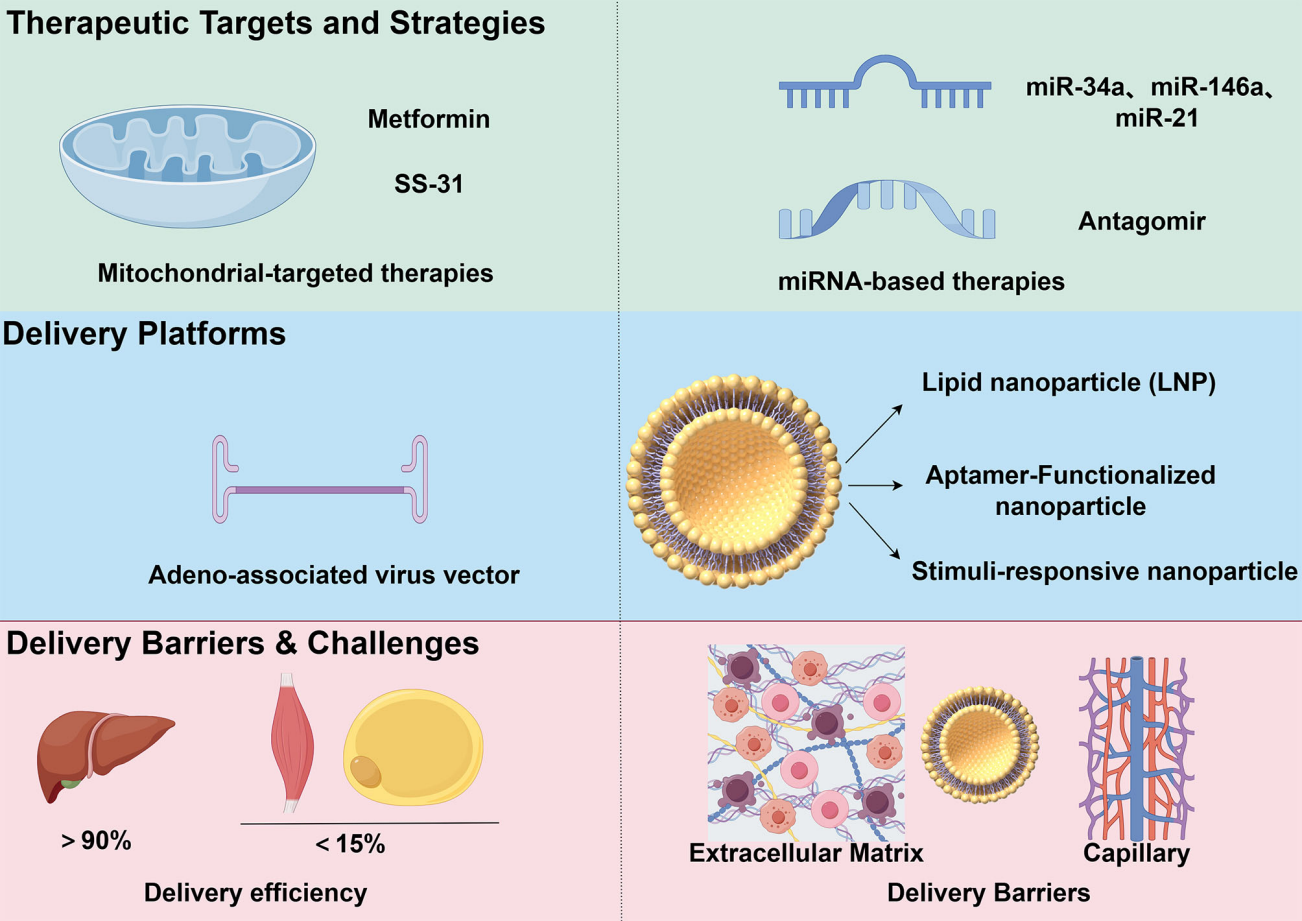
Therapeutic strategies and delivery barriers targeting the mitochondrial–ROS–miRNA axis. Interventions include mitochondria-targeted agents and miRNA-based strategies. Metformin is highlighted as a multi-modal agent: partial Complex I inhibition is widely supported, yet additional mechanisms are reported, including AMPK activation via cellular energy/redox shifts, effects on the mitochondrial glycerophosphate shuttle, and lysosome-linked signaling pathways. SS-31 stabilizes cardiolipin and respiratory supercomplexes.miRNA modalities such as antagomir-34a and miR-146a mimics. Emerging delivery vehicles—LNPs, AAVs, aptamer-guided carriers and stimulus-responsive nanoparticles—achieve modest extrahepatic exposure, typically under about 15% in muscle or adipose tissue, and contend with barriers imposed by the extracellular matrix and capillary endothelium.

Mitochondrial dysfunction and oxidative stress have been increasingly recognized as potential therapeutic targets for T2DM. Recent research advances have driven the development of two major therapeutic strategies: (1) pharmacological agents directly modulating mitochondrial bioenergetics, and (2) miRNA-based interventions aimed at restoring redox homeostasis and insulin sensitivity. However, the clinical translation of these therapies is constrained by multiple factors, including insufficient delivery efficiency to insulin-sensitive tissues and complex regulatory feedback loops involving ROS and miRNAs. A comprehensive understanding of therapeutic modalities, delivery technologies, and relevant biological barriers is crucial for optimizing intervention strategies. **Figure 3** systematically integrates current therapeutic strategies targeting mitochondria and miRNAs, representative delivery platforms, and key physiological obstacles limiting their efficacy.

Interventions include mitochondria-targeted agents and miRNA-based strategies. Metformin is highlighted as a multi-modal agent: partial Complex I inhibition is widely supported, yet additional mechanisms are reported, including AMPK activation via cellular energy/redox shifts, effects on the mitochondrial glycerophosphate shuttle, and lysosome-linked signaling pathways. SS-31 stabilizes cardiolipin and respiratory supercomplexes.miRNA modalities such as antagomir-34a and miR-146a mimics. Emerging delivery vehicles—LNPs, AAVs, aptamer-guided carriers and stimulus-responsive nanoparticles—achieve modest extrahepatic exposure, typically under about 15% in muscle or adipose tissue, and contend with barriers imposed by the extracellular matrix and capillary endothelium.

### 6.1 Mitochondria-targeted pharmacological strategies

As a first-line therapeutic agent for T2DM, metformin primarily exerts its effects through reversible inhibition of mitochondrial electron transport chain complex I (ETC-I), thereby reducing OXPHOS efficiency (102, 103). The subsequent increase in the AMP/ATP ratio activates AMP-activated protein kinase (AMPK), leading to enhanced glucose uptake and fatty acid oxidation, ultimately improving peripheral insulin sensitivity (104, 105). However, chronic ETC-I inhibition may trigger compensatory metabolic adaptations that could limit its long-term efficacy. While partial Complex I inhibition with secondary AMPK activation remains the leading model for metformin, alternative or complementary mechanisms-including mitochondrial/glycerophosphate shuttle effects, redox-linked signaling, and non-mitochondrial kinase modulation-have been reported. These nuances do not negate the Complex I paradigm but qualify it as predominant rather than exclusive.Metabolic reprogramming: Prolonged ETC-I inhibition stimulates glycolytic metabolism as a compensatory mechanism for ATP production, resulting in lactate accumulation (106)—a phenomenon particularly detrimental to patients with renal impairment. Impaired antioxidant capacity: ETC-I inhibition reduces the generation of reduced coenzyme Q10 (CoQH_2_), compromising mitochondrial ROS scavenging capacity and increasing susceptibility to oxidative stress (107, 108). SS-31, an aromatic cationic mitochondria-targeted peptide, selectively binds to cardiolipin on the mitochondrial inner membrane (109). ETC supercomplex stabilization: SS-31 preserves the integrity of ETC supercomplexes, reducing electron leakage and mitochondrial O_2_^˙−^ generation (110–112). Cardiolipin protection: By maintaining cardiolipin conformation, SS-31 prevents cytochrome c dissociation from the inner membrane, ensuring efficient ATP synthesis (113, 114). However, the clinical application of SS-31 may be limited due to its heterogeneous tissue penetration and suboptimal half-life. In addition to pharmacological agents, emerging biomaterial-based strategies have shown promise in restoring mitochondrial homeostasis in diabetic contexts. For instance, a bioenergetically-active poly(glycerol sebacate)-based multiblock hydrogel has been demonstrated to promote diabetic wound healing by revitalizing mitochondrial metabolism, improving local ATP production, and reducing oxidative stress levels (115). Although primarily applied in regenerative medicine, the mechanism—enhancing mitochondrial bioenergetics and redox balance—is fundamentally aligned with efforts to counteract insulin resistance.Such approaches offer conceptual evidence for extending mitochondrial-targeted therapy beyond classical drug frameworks, especially in combination with systemic treatments.

### 6.2 miRNA-based therapeutic interventions

In insulin-sensitive tissues of patients with T2DM, the expression of miR-34a is markedly upregulated, contributing to mitochondrial dysfunction and impaired metabolic homeostasis (116, 117). This upregulation reduces NAD^+^ levels by targeting SIRT1 and NAMPT, subsequently suppressing SIRT1 activity and impairing mitochondrial function (118). miR-146a has emerged as a key protective miRNA against oxidative stress and chronic inflammation in the diabetic milieu. It exerts its beneficial effects by negatively regulating the TRAF6/NF-κB signaling axis and suppressing NOX4-mediated ROS generation. TRAF6 inhibition: miR-146a blocks IKKβ-mediated IRS-1 serine 307 phosphorylation by suppressing TRAF6, restoring insulin signaling integrity (119, 120). NOX4 suppression: NOX4 downregulation reduces cytosolic ROS production by ~50%, subsequently alleviating mitochondrial oxidative stress (121, 122). Exosome-based miR-146a delivery has shown insulin-sensitizing efficacy *in vivo*, characterized by NF-κB suppression, decreases in pro-inflammatory cytokines including IL-1β and IL-18, and normalization of tissue redox status (123). Such findings suggest that exosomal miR-146a represents a promising non-pharmacological strategy for modulating mitochondrial stress responses in T2DM. In addition to direct miRNA-targeted interventions, several naturally derived compounds have demonstrated miRNA-modulatory properties and beneficial effects on mitochondrial function and oxidative stress. Cannabidiol (CBD), a non-psychoactive phytocannabinoid, has shown anti-inflammatory and antioxidant activity in diabetic models. Its effects may be mediated through inhibition of mitochondrial ROS production, suppression of NLRP3 inflammasome activation, and activation of AMPK signaling, thereby improving insulin sensitivity and protecting β-cell function. These findings suggest that CBD could serve as a complementary agent alongside miRNA-targeted therapies in the treatment of metabolic disorders such as T2DM (124).

## 7 Challenges and future directions

Mitochondrial bioenergetics and miRNA-mediated redox feedback form a multilayered network in insulin-sensitive tissues. Their crosstalk sustains insulin resistance through diminished oxidative capacity, persistent oxidative distress, and cell-type–specific vulnerability. Current delivery technologies enrich the liver yet show limited exposure in skeletal muscle and adipose tissue, often below fifteen percent, with additional barriers from extracellular matrix and endothelial interfaces (125–128). Beyond descriptive studies, the field still lacks integrative, quantitative models that couple metabolic flux, ROS dynamics, and miRNA regulation across organs. Neuro-metabolic involvement is increasingly evident; altered hippocampal oscillations and cortico-hippocampal desynchrony in type 2 diabetes point to brain–metabolism crosstalk that merits systems-level treatment (129). Cross-tissue miRNA signaling that influences the PPARγ axis further underscores the need for human tissue profiling, longitudinal cohorts, and harmonized assays to quantify effect sizes and tissue specificity (130).Human tissue profiling, longitudinal cohorts, and standardized assays are required to define the effect sizes and tissue specificity of candidate miRNAs in T2DM.

Translational progress will depend on two tracks. First, platform innovation: dual-targeting aptamers and microenvironment-responsive nanoparticles can improve extrahepatic delivery and reduce off-target exposure, while advances in biofabrication offer spatially programmable depots for localized mitochondrial and redox modulation in diabetic tissues (131). Second, analytic integration: multi-organ modeling that unifies mitochondrial metabolism, redox kinetics, and miRNA control should support in-silico stratification and prospective trial design. Circulating miRNAs remain promising dynamic biomarkers of oxidative stress and therapeutic response; assay standardization and clinical thresholds are required for deployment in precision care (132–134).

Looking ahead, miRNA-directed interventions are best positioned as adjuncts to mitochondrial therapies. Priority steps include selecting tissue-enriched targets with human validation, developing delivery systems with verified extrahepatic uptake, establishing pharmacodynamic reporters that track redox correction *in vivo*, and building companion diagnostics that combine circulating miRNAs with metabolic imaging. Together, these steps can convert the mitochondria–ROS–miRNA framework into testable clinical strategies.
